# Pediatric and adolescent HIV viral load coverage and suppression rates in the context of the COVID-19 pandemic in 12 PEPFAR-supported sub-Saharan African countries in 2019 and 2020

**DOI:** 10.1371/journal.pgph.0003513

**Published:** 2024-08-01

**Authors:** Deborah Carpenter, Marisa Hast, Nicole Buono, Susan Hrapcak, Kimi Sato, Rosemary Mrina, Mackenzie Hurlston Cox, Patricia Aladi Agaba, Alexandra C. Vrazo, Hilary Wolf, Emilia D. Rivadeneira, Judith D. Shang, Magdalene Mange Mayer, Aka Herve Prao, Henri Onema Longuma, Constantin Kabwe, Patricia Nyembo Lwana, Tsegaye Tilahun, Mamorapeli Ts’oeu, Immaculate Mutisya, Lennah Nyabiage Omoto, Jessica Greenberg Cowan, Maria Ines Jorge Tomo de Deus, Omodele Johnson Fagbamigbe, Uzoma Ene, Akudo Ikpeazu, Mduduzi B. Ndlovu, Eva Matiko, Nicolas Schaad, Jema Bisimba, Elizabeth Lema, Kebby Musokotwane, Talent Maphosa, Buyile Buthelezi, Adegbenga Olarinoye, Ismail Lawal, Solomon Mukungunugwa, Janet Tulibonywa Mwambona, Teferi Wondimu, Immaculate Anne Kathure, Onyeka Donald Igboelina, Valery Nxima Nzima, Rosine Grace Bissai, Matjeko Lenka, Willibrord Shasha, N’guetta-Kan Olivier, Mѐrcia Matsinhe, Argentina Wate, Lingenda Godfrey, Heather Alexander, George Alemnji, Shirley Lecher

**Affiliations:** 1 Division of Global HIV and Tuberculosis, Center for Global Health, Centers for Disease Control and Prevention, Atlanta, Georgia; 2 United States Agency for International Development, Washington, District of Columbia, United States of America; 3 Walter Reed Army Institute of Research, United States Military HIV Research Program, Silver Spring, Maryland, United States of America; 4 The Henry M. Jackson Foundation for the Advancement of Military Medicine, Bethesda, Maryland, United States of America; 5 US Department of State, Office of the United States Global AIDS Coordinator, Washington, District of Columbia, United States of America; 6 Division of Global HIV and Tuberculosis, Center for Global Health, Centers for Disease Control and Prevention, Yaounde, Cameroon; 7 Division of Global HIV and Tuberculosis, Center for Global Health, Centers for Disease Control and Prevention, Abidjan, Côte d’Ivoire; 8 Division of Global HIV and Tuberculosis, Center for Global Health, Centers for Disease Control and Prevention, Kinshasa, Democratic Republic of Congo; 9 Ministry of Health, National AIDS Control Committee, Kinshasa, Democratic Republic of Congo; 10 United States Agency for International Development, Addis Ababa, Ethiopia; 11 Division of Global HIV and Tuberculosis, Center for Global Health, Centers for Disease Control and Prevention, Maseru, Lesotho; 12 Division of Global HIV and Tuberculosis, Center for Global Health, Centers for Disease Control and Prevention, Nairobi, Kenya; 13 Division of Global HIV and Tuberculosis, Center for Global Health, Centers for Disease Control and Prevention, Maputo, Mozambique; 14 Division of Global HIV and Tuberculosis, Center for Global Health, Centers for Disease Control and Prevention, Nigeria; 15 Federal Ministry of Health, Abuja, Nigeria; 16 Division of Global HIV and Tuberculosis, Center for Global Health, Centers for Disease Control and Prevention, Pretoria, South Africa; 17 Division of Global HIV and Tuberculosis, Center for Global Health, Centers for Disease Control and Prevention, Dar es Salaam, Tanzania; 18 United States Agency for International Development, Dar es Salaam, Tanzania; 19 Division of Global HIV and Tuberculosis, Center for Global Health, Centers for Disease Control and Prevention, Lusaka, Zambia; 20 Division of Global HIV and Tuberculosis, Center for Global Health, Centers for Disease Control and Prevention, Harare, Zimbabwe; 21 United States Agency for International Development, Pretoria, South Africa; 22 HJF Medical Research International, Abuja, Nigeria; 23 US Army Medical Research Directorate-Africa/ Walter Reed Army Institute of Research, Abuja, Nigeria; 24 United States Agency for International Development, Harare, Zimbabwe; 25 Walter Reed Army Institute of Research (WRAIR), Dar es Salaam, Tanzania; 26 Division of Global HIV and Tuberculosis, Center for Global Health, Centers for Disease Control and Prevention, Addis Ababa, Ethiopia; 27 United States Agency for International Development, Nairobi, Kenya; 28 United States Agency for International Development, Abuja, Nigeria; 29 United States Agency for International Development, Yaounde, Cameroon; 30 United States Agency for International Development, Maseru, Lesotho; 31 United States Agency for International Development, Abidjan, Côte d’Ivoire; 32 United States Agency for International Development, Maputo, Mozambique; 33 United States Agency for International Development, Lusaka, Zambia; GU: Georgetown University, UNITED STATES OF AMERICA

## Abstract

The early period of the COVID-19 pandemic limited access to HIV services for children and adolescents living with HIV (C/ALHIV). To determine progress in providing care and treatment services, we describe viral load coverage (VLC) and suppression (VLS) (<1000 copies/ mL) rates during the COVID-19 pandemic in 12 United States President’s Emergency Plan for AIDS Relief (PEPFAR)-supported countries. Data for children (0–9 years) and adolescents (10–19 years) on VLC and VLS were analyzed for 12 sub-Saharan African (SSA) countries between 2019 (pre-COVID-19) and 2020 (during COVID-19). We report the number of viral load (VL) tests, and percent change in VLC and VLS for patients on ART. For 12 countries, 181,192 children had a VL test during the pre-COVID-19 period compared with 177,683 December 2020 during COVID-19. VLC decreased from 68.8% to 68.3% overall. However, 9 countries experienced an increase ranging from a 0.7%-point increase for Tanzania and Zimbabwe to a 15.3%-point increase for Nigeria. VLS increased for all countries from 71.2% to 77.7%. For adolescents the number with a VL test increased from 377,342 to 402,792. VLC decreased from 77.4% to 77.1%. However, 7 countries experienced an increase ranging from 1.8% for Mozambique to 13.8% for Cameroon. VLS increased for all countries from 76.8% to 83.8%. This analysis shows variation in HIV VLC across 12 SSA countries. VLS consistently improved across all countries demonstrating resilience of countries during 2020. Countries should continue to improve clinical outcomes from C/ALHIV despite service disruptions that may occur during pandemic response.

## Introduction

The beginning of the COVID-19 pandemic stretched healthcare systems, limiting access to HIV services for children and adolescents living with HIV (C/ALHIV) [1,2]. Globally, there were 2.7 million C/ALHIV in 2021, with approximately nine out of ten residing in Sub-Saharan Africa (SSA) [3,4]. Substantial gains in HIV prevention and treatment have occurred. According to the UNAIDS Global Report for 2023, the numbers of new child HIV infections decreased from 287,000 in 2010 to 109,000 in 2022 which accounts for 94% of all child HIV infections averted globally through vertical transmission programs [5]. New HIV infections among children <15 years old dropped 52%, from 320,000 in 2010 to 160,000 in 2021 [6]. The number of deaths from HIV related illnesses have also declined in children <15 years old by 59%, from 240,000 in 2010 to 98,000 in 2021 [7]. Among adolescents 10–19 years old living with HIV (ALHIV), there was a 36% reduction in new HIV infections, from 250,000 in 2010 to 160,000 in 2021. Likewise, the number of AIDS-related deaths among ALHIV declined 37% (from 51,000 in 2010 to 29,000 in 2021) [7]. Despite this progress, the greatest burden of C/ALHIV is in SSA, and acquired immunodeficiency syndrome (AIDS) is a major cause of mortality among C/ALHIV [8]. Syndemic interactions between COVID-19 and HIV created barriers to accessing essential HIV services, threatening to reverse the HIV clinical and programmatic gains made in recent years [9–11]. During March-April 2020, Ministries of Health in collaboration with the United States President’s Emergency plan for AIDS Relief (PEPFAR) and the Global Fund to Fight AIDS, Tuberculosis, and Malaria, issued guidance to adapt HIV programs to facilitate continuity of HIV treatment for vulnerable populations, including C/ALHIV during the COVID-19 pandemic [12,13].

The World Health Organization (WHO) recommends that all C/ALHIV receive antiretroviral treatment (ART) and are monitored six and twelve months after ART initiation and annually thereafter using HIV viral load (VL) to monitor treatment. C/ALHIV in all PEPFAR countries receive WHO recommended ART regimens. VL coverage (VLC) has been lower for children 0–9 years old (58%) compared to ALHIV (82%) and adults 20+ years living with HIV (77%) as of June 30, 2021 [14]. VL monitoring is an essential service for C/ALHIV especially those with virologic failure who are at risk of suboptimal clinical outcomes. Strategic adaptations to improve VL testing during future public health emergencies may be necessary to maintain VL monitoring and to inform treatment effectiveness and success [2,15].

Achieving Viral Load Suppression (VLS) for all people living with HIV (PLHIV) is a global priority and a key measure of treatment efficacy [16]. An unsuppressed VL is associated with higher morbidity and mortality [17]. Many countries in SSA are not on track to achieve the UNAIDS third 95 target of 95% of CLHIV on ART attaining viral suppression by 2030 [18]. For PEPFAR countries, VLS for children 0–9 years (77%) and adolescents 10–19 years old (85%) are lower than adults 20+ years old living with HIV (94%) [14].

Barriers to VL testing and VL suppression in C/ALHIV include challenges with sample collection, caregivers or parents not bringing children for appointments and challenges with ARV formulations and adherence [19,20]. C/ALHIV are at higher risk for morbidity and mortality and continue to be disproportionately affected by HIV/AIDS. Each day in 2021, approximately 850 children became infected with HIV and approximately 301 children died from AIDS related causes [4].

This analysis describes changes in VLC and VLS in C/ALHIV from January 2019 (pre-COVID-19) through December 2020 (during COVID-19) in 12 PEPFAR SSA countries to help inform decisions and develop strategies for programmatic improvement during COVID-19 and future public health threats.

## Methods

Data for this analysis was used from standardized PEPFAR monitoring evaluation and reporting (MER) surveillance datasets. The MER data include a routine set of pre-defined indicators that are reported quarterly as aggregate data by each health facility and then are entered by each PEPFAR country office to a centralized cloud-based reporting system. Quarters in PEPFAR are defined according to US government fiscal year as (Q1) October 1-December 31; (Q2) January 1-March 31; (Q3) April 1-June 30; and (Q4) July 1-September 30. For each quarter of PEPFAR reporting, submitted data covering the defined time period are then cleaned and reviewed by the State Department Office of the Global AIDS Coordinator and are released to be available online as the structured downloadable MER dataset [14]. For this analysis, data were downloaded for the period from January 2019 through December 2020, which corresponds with 8 quarterly reporting periods.

For all analyses, data were examined for children (0–9 years) and adolescents (10–19 years) separately in 12 countries: Cameroon, Côte d’Ivoire, Democratic Republic of the Congo (DRC), Ethiopia, Lesotho, Kenya, Mozambique, Nigeria, South Africa, Tanzania, Zambia, and Zimbabwe. These 12 countries were selected among PEPFAR countries, for their high HIV burden and large pediatric treatment gaps. Globally these countries are among the 16 countries accounting for 90% of the pediatric ART coverage gap [5]. To describe the potential effect of the COVID-19 pandemic on VL in these countries, the following PEPFAR VL indicators were used:

1. Number of patients at the end of the quarterly reporting period who were eligible for a VL test, defined as the total number who have been on ART for ≥ 6 months;
2. Number of patients at the end of the quarterly reporting period who had any documented VL result in the medical or laboratory record in the past 12 months;
3. Number of patients at the end of the quarterly reporting period who had a documented VL test in the past 12 months with suppressed VL (defined as VL under 1000 copies/mL) at their most recent VL test;
4. Percent VLC, calculated as number of patients with VL test results in the past 12 months (Indicator 2) divided by total number of patients eligible for a VL test (Indicator 1) at end of the quarterly reporting period; and
5. Percent VLS, calculated as the number of patients with suppressed VL (Indicator 3) divided by the number of patients with VL test results in the past 12 months (Indicator 2) at the end of the quarterly reporting period [21].

For each of these indicators, the absolute and percent change was calculated by age group between data reported in December 2019 (FY2020 Q1) and December 2020 (FY2021 Q1). Since PEPFAR VL indicators by definition provide VL information for patients over the previous 12 months as described above, this calculation effectively compares each VL metric for the full year of 2019 to the full year of 2020. Trends over the reporting period were calculated overall, by sex, and by individual country. Graphics were developed to visualize the change in indicators over each quarter of data by age group and to visualize the change in VLC and VLS by country over the two time points. All analyses were conducted in Microsoft Excel Query and Microsoft Excel, Office 365 version (Redmond, WA).

Ethical Considerations: This project was reviewed in accordance with United States Centers for Prevention and Disease Control and United States Agency for International Development human research protection procedures and was determined to be non-research. The data used for this analysis contained no personally identifiable information. Only surveillance data were used for this analysis.

## Results

### Children 0–9 years VL testing, coverage, and suppression

The total number of children 0–9 years on ART eligible for VL testing from 2019 to 2020 for all 12 countries was reduced by 1.2%, (263,332 to 260,237) coinciding with a 1.9% decrease in the total number of children receiving a VL test (181,192 to 177,683) (Table 1). The decline in the total number eligible for a VL test resulted from a decrease in six countries, from the largest number of children in South Africa (50,567 to 45,989), followed by Mozambique (43,025 to 42,428), Kenya (35,902 to 32,239), Ethiopia (10,352 to 7,353), Côte d’Ivoire (6,949 to 5,799) and Lesotho (3,835 to 3,250) (Table 1). The remaining six countries reported increases in the number of children eligible for VL testing. The decline in the total number of VL tests reported was driven by five of the six countries which also reported a decline in the number of children eligible for a VL test, South Africa (39,145 to 30,730), Kenya (33,613 to 28,439), Ethiopia (6,367 to 4,143), Côte d’Ivoire (5,164 to 4,561) and Lesotho (2,825 to 2,481) (Table 1). Only Mozambique reported a decrease in the number of children eligible for a VL test but an increase in the number of children that received a VL test (24,809 to 26,276) (Table 1). The numbers of children with VL suppression improved by 7.0% (129,054 to 138,060) (Table 1). Nine countries reported improvement in numbers of children with suppressed VL ranging from 0.1% (2,415 to 2,418) for Lesotho to 121% (1,141 to 2,524) for Cameroon, while 3 countries South Africa 21.3% (30,674 to 24,138), Kenya 11.0% (26,920 to 23,963) and Ethiopia 22.7% (4,437 to 3,432) reported a decline (Table 1).

**Table 1:** Change in number eligible for viral load (VL) testing, number who received VL results, and number virally suppressed among children and adolescents in 12 PEPFAR programs, 2019 (FY2020 Q1) to 2020 (FY2021 Q1)*.

Eligible for VL Testing	Children (0 to 9)			Adolescents (10 to 19)		
	2019	2020	% Change	2019	2020	% Change
Cameroon	3,954	6,347	+60.5%	6,089	9,140	+50.1%
Cote d’Ivoire	6,949	5,799	-16.5%	8,124	7,895	-2.8%
DRC	5,416	7,532	+39.1%	5,699	7,849	+37.7%
Ethiopia	10,352	7,353	-29.0%	26,356	25,907	-1.7%
Kenya	35,902	32,239	-10.2%	71,970	74,170	+3.1%
Lesotho	3,835	3,250	-15.3%	9,683	9,276	-4.2%
Mozambique	43,025	42,428	-1.4%	46,467	47,299	+1.8%
Nigeria	21,060	22,921	+8.8%	28,268	36,298	+28.4%
South Africa	50,567	45,989	-9.1%	131,752	132,946	+0.9%
Tanzania	33,357	33,664	+0.9%	52,706	56,693	+7.6%
Zambia	23,139	24,239	+4.8%	45,749	50,283	+9.9%
Zimbabwe	25,776	28,476	+10.5%	54,490	64,813	+18.9%
Total	263,332	260,237	-1.2%	487,353	522,569	+7.2%
**# w/VL Results**						
Cameroon	1,904	3,778	+98.4%	3,240	6,126	+89.1%
Côte d’Ivoire	5,164	4,561	-11.7%	7,243	6,771	-6.5%
DRC	2,801	4,724	+68.7%	3,595	5,901	+64.1%
Ethiopia	6,367	4,143	-34.9%	22,419	19,647	-12.4%
Kenya	33,613	28,439	-15.4%	71,384	71,351	-0.0%
Lesotho	2,825	2,481	-12.2%	7,728	7,885	+2.0%
Mozambique	24,809	26,276	+5.9%	29,515	30,874	+4.6%
Nigeria	14,080	18,837	+33.8%	23,112	33,230	+43.8%
South Africa	39,145	30,730	-21.5%	102,784	99,262	-3.4%
Tanzania	24,367	24,818	+1.9%	44,606	46,497	+4.2%
Zambia	15,372	16,838	+9.5%	33,037	39,385	+19.2%
Zimbabwe	10,745	12,058	+12.2%	28,679	35,863	+25.0%
Total	181,192	177,683	-1.9%	377,342	402,792	+6.7%
**# VL Suppressed**						
Cameroon	1,141	2,524	+121.2%	1,949	4,429	+127.2%
Côte d’Ivoire	3,063	3,203	+4.6%	4,385	4,989	+13.8%
DRC	2,190	4,178	+90.8%	2,798	5,273	+88.5%
Ethiopia	4,437	3,432	-22.7%	17,863	17,385	-2.7%
Kenya	26,920	23,963	-11.0%	58,264	62,680	+7.6%
Lesotho	2,415	2,418	+0.1%	6,838	8,257	+20.8%
Mozambique	11,627	15,852	+36.3%	18,751	24,332	+29.8%
Nigeria	8,611	14,423	+67.5%	16,063	27,706	+72.5%
South Africa	30,674	24,138	-21.3%	82,049	79,812	-2.7%
Tanzania	18,269	20,818	+14.0%	34,327	40,343	+17.5%
Zambia	11,787	13,907	+18.0%	25,214	34,039	+35.0%
Zimbabwe	7,920	9,204	+16.2%	21,431	28,115	+31.2%
Total	129,054	138,060	+7.0%	289,932	337,360	+16.4%

*FY2020 Q1 captures VL data in the past 12 months at the end of December 2019. FY2021 Q1 captures VL data in the past 12 months at the end of December 2020.

For all countries there was a slight 0.5% decrease in VLC for children (Table 2). The majority of countries experienced an increase in VLC during this time period, ranging from 0.7% for Tanzania to 11.4% for Cameroon. Three countries Ethiopia, Kenya and South Africa reported a decline in VLC of 5.2%, 5.4% and 10.6% respectively. Only two countries, Nigeria and Kenya reported VLC > 80%.

**Table 2:** Viral load coverage (VLC) and viral load suppression (VLS) among children and adolescents by country across two time points: 2019 (FY2020 Q1) to 2020 (FY2021 Q1) in 14 PEPFAR programs*.

Children (Age 0–9)	**VLC (%)			***VLS (%)		
	2019	2020	Point change	2019	2020	Point change
Cameroon	48.2	59.5	+11.4	59.9	66.8	+6.9
Cote d’Ivoire	74.3	78.7	+4.3	59.3	70.2	+10.9
DRC	51.7	62.7	+11.0	78.2	88.4	+10.3
Ethiopia	61.5	56.3	-5.2	69.7	82.8	+13.2
Kenya	93.6	88.2	-5.4	80.1	84.3	+4.2
Lesotho	73.7	76.3	+2.7	85.5	97.5	+12.0
Mozambique	57.7	61.9	+4.3	46.9	60.3	+13.5
Nigeria	66.9	82.2	+15.3	61.2	76.6	+15.4
South Africa	77.4	66.8	-10.6	78.4	78.5	+0.2
Tanzania	73.0	73.7	+0.7	75.0	83.9	+8.9
Zambia	66.4	69.5	+3.0	76.7	82.6	+5.9
Zimbabwe	41.7	42.3	+0.7	73.7	76.3	+2.6
Total	68.8	68.3	-0.5	71.2	77.7	+6.5
**Adolescents (Age 10–19)**						
Cameroon	53.2	67.0	+13.8	60.2	72.3	+12.1
Côte d’Ivoire	89.2	85.8	-3.4	60.5	73.7	+13.1
DRC	63.1	75.2	+12.1	77.8	89.4	+11.5
Ethiopia	85.1	75.8	-9.2	79.7	88.5	+8.8
Kenya	99.2	96.2	-3.0	81.6	87.8	+6.2
Lesotho	79.8	85.0	+5.2	88.5	104.7***	+16.2
Mozambique	63.5	65.3	+1.8	63.5	78.8	+15.3
Nigeria	81.8	91.5	+9.8	69.5	83.4	+13.9
South Africa	78.0	74.7	-3.3	79.8	80.4	+0.6
Tanzania	84.6	82.0	-2.6	77.0	86.8	+9.8
Zambia	72.2	78.3	+6.1	76.3	86.4	+10.1
Zimbabwe	52.6	55.3	+2.7	74.7	78.4	+3.7
Total	77.4	77.1	-0.3	76.8	83.8	+6.9

*FY2020 Q1 captures VL data in the past 12 months at the end of December 2019. FY2021 Q1 captures VL data in the past 12 months at the end of December 2020

**VLC rates calculated as number of patients with VL results divided by total number of patients eligible for a VL test (on ART ≥ 6 months). **VLS rates, calculated as the number of patients with VL below 1,000 copies/mL divided by the number of patients with VL results

*** VLC result of >100% may be due to data quality challenges, incomplete or under-reporting of patients current on ART. Lack of unique identifiers and EMR may result in double counting of VL results per patient.

Results for children 0–9 years by gender show a minimal difference for boys and girls eligible for VL testing (a 1.3% and a 1.1% decrease respectively) and with VL results (a 1.6% and a 2.3% decrease respectively) (Table 3). Boys had a higher increase of 8.4% in number with VL suppressed results (5,127) compared to girls 5.7% increase (3,879) from 2019 to 2020. Minimal difference is also seen for boys and girls on VLC (a 0.3% and 1.2% decrease respectively) (Table 3).

**Table 3:** Absolute and percent change in number eligible for viral load (VL) testing, number who received VL results, number virally suppressed, percent viral load coverage (VLC*), and percent viral load suppression (VLS**) among children and adolescents in 14 PEPFAR programs, 2019 (FY2020 Q1) to 2020 (FY2021 Q1)*.

Eligible for VL Testing	Children (0 to 9)				Adolescents (10 to 19)			
	2019	2020	Absolute change	% Change	2019	2020	Absolute change	% Change
Girls	136,243	134,744	-1,499	-1.1%	284,624	302,272	+17,648	+6.2%
Boys	127,089	125,493	-1,596	-1.3%	202,729	220,297	+17,568	+8.7%
Total	263,332	260,237	-3,095	-1.2%	487,353	522,569	+35,216	+7.2%
**# w/VL Results**								
Girls	93,905	91,771	-2,134	-2.3%	213,273	225,206	+11,933	+5.6%
Boys	87,287	85,912	-1,375	-1.6%	164,069	177,586	+13,517	+8.2%
Total	181,192	177,683	-3,509	-1.9%	377,342	402,792	+25,450	+6.7%
**# VL Suppressed**								
Girls	68,340	72,219	+3,879	+5.7%	167,298	190,307	+23,009	+13.8%
Boys	60,714	65,841	+5,127	+8.4%	122,634	147,053	+24,419	+19.9%
Total	129,054	138,060	+9,006	+7.0%	289,932	337,360	+47,428	+16.4%
*VLC %								
Girls	68.9	68.1	-0.8	-1.2%	74.9	74.5	-0.4	-0.6%
Boys	68.7	68.5	-0.2	-0.3%	80.9	80.6	-0.3	-0.4%
Total	68.8	68.3	-0.5	-0.8%	77.4	77.1	-0.3	-0.4%
** *VLS %*								
Girls	72.8	78.7	+5.9	+8.1%	78.4	84.5	+6.1	+7.7%
Boys	69.6	76.6	+7.1	+10.2%	74.7	82.8	+8.1	+10.8%
Total	71.2	77.7	+6.5	+9.1%	76.8	83.8	+6.9	+9.0%

*FY2020 Q1 captures VL data in the past 12 months at the end of December 2019. FY2021 Q1 captures VL data in the past 12 months at the end of December 2020.

**VLC rates calculated as the number of patients with VL results divided by the total number of patients eligible for a VL test (on ART ≥ 6 months)

***VLS rates, calculated as the number of patients with VL below 1,000 copies/mL divided by the number of patients with VL results.

 

### Adolescents 10–19 years VL testing, coverage, and suppression

In contrast to children, the number of adolescents eligible for VL testing increased 7.2% (487,353 to 522,569) from 2019 to 2020 coinciding with an overall increase in the number of adolescents with a VL test of 6.7% (377,342 to 402,792) (Table 1). The increase in number of adolescents eligible for a VL test for nine countries ranged from 0.9% (131,752 to 132,946) for South Africa to 50.1% (6,089 to 9,140) for Cameroon (Table 1). Three countries reported a decrease in the number of adolescents eligible for a VL test, Lesotho 4.2% (9,683 to 9,276), Côte d’Ivoire 2.8% (8,124 to 7,895) and Ethiopia 1.7% (26,356 to 25,907). Eight countries reported an increase in the number of adolescents with VL tests, ranging from the lowest Lesotho with 2.0% (7,728 to 7,885) to the highest Cameroon with 89.1% (3,240 to 6,126) (Table 1). Four countries reported a decline in the number of VL tests for adolescents, South Africa 3.4% (102,784 to 99,262), Ethiopia 12.4% (22,419 to 19,647), Côte d’Ivoire 6.5% (7,243 to 6,771) and Kenya reported a minimal decrease <1% (71,384 to 71,351) Table1. The numbers of adolescents with VL suppression increased by 16.4% (289,932 to 337,360). Ten countries reported improvement in numbers of adolescents with suppressed VL, ranging from 7.6% (58,264 to 62,680) for Kenya to 127.2% (1,949 to 4,429) for Cameroon, while two countries South Africa 2.7% (82,049 to 79,812) and Ethiopia 2.7% (17,863 to 17,385) reported a decline (Table 1).

**Fig 1:**
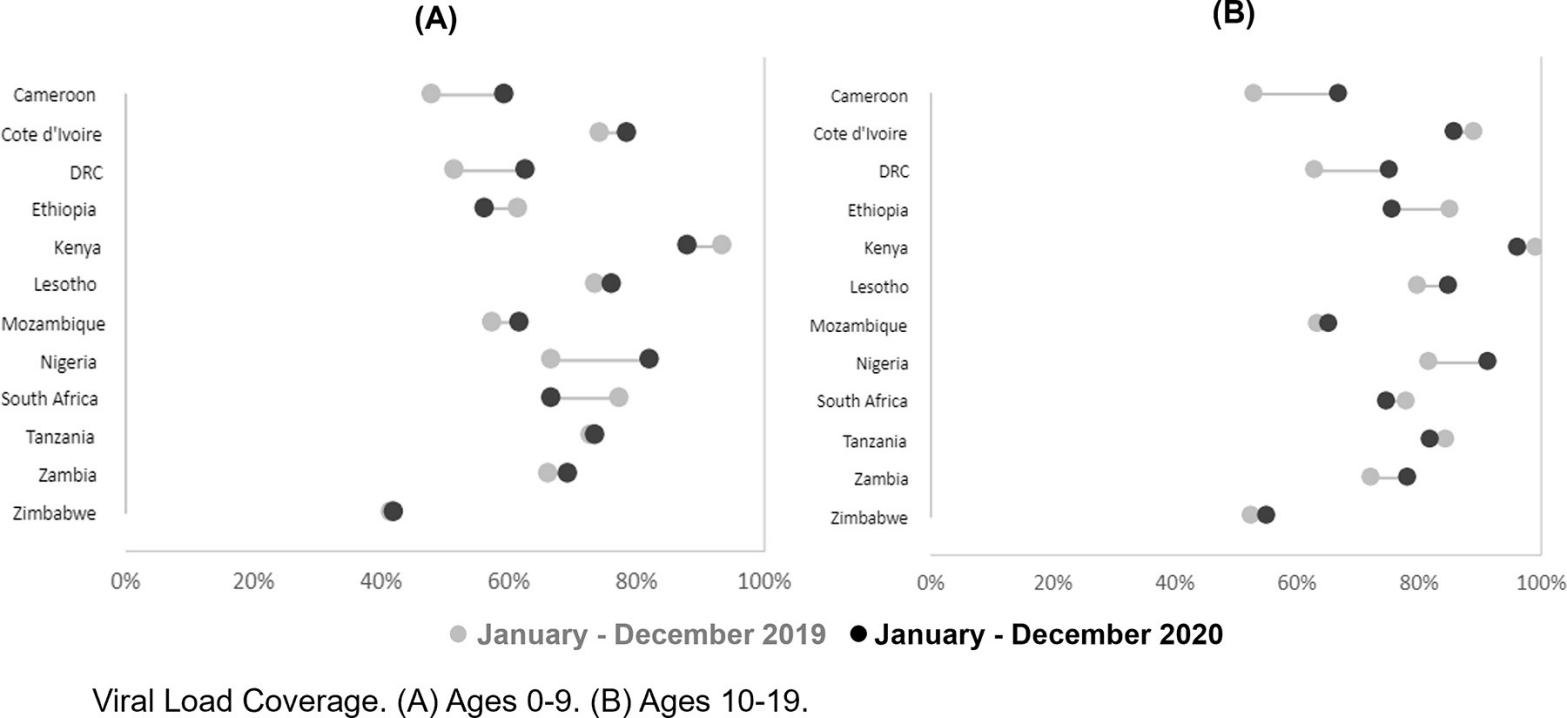
Change in viral load coverage (VLC) for children 0–9 years and adolescents 10–19 years between December 2019 and December 2020. The lighter dot denotes December 2019, and the darker color denotes December 2020. For each graph programmatic VLC the UNAIDS/WHO/PEPFAR goal is 95%.

For all countries there was a slight 0.3% decrease in VLC for adolescents. This decrease was driven by five countries reporting a reduction in VLC, Tanzania 2.6%, Kenya 3.0%, South Africa 3.3%, Côte d’Ivoire 3.4%, and Ethiopia 9.2% (Table 2 and Fig 1). Seven countries experienced an increase in VL coverage ranging from the lowest Mozambique 1.8%, Zimbabwe 2.7%, Lesotho 5.2%, Zambia 6.1%, Nigeria 9.8%, DRC 12.1%, to the highest Cameroon 13.8% (Table 2). For adolescents, 5 countries, Côte d’Ivoire, Kenya, Lesotho, Nigeria, and Tanzania reported >80% VLC. The goal of 95% VLC for adolescents was achieved only by Kenya.

Results by gender show that adolescent boys 10–19 years had an increase of 8.7% in the number eligible for VL testing and an increase of 8.2% in the number with VL results compared to a 6.2% and a 5.6% increase for adolescent girls from 2019 to 2020 (Table 3). More adolescent boys experienced an increase in viral suppression 24,419 compared to girls 23,009 (Table 3). Minimal difference is also seen for adolescent boys and girls on VLC (a 0.4% and 0.6% decrease respectively) (Table 3).

## Discussion

This analysis demonstrated a small overall decline in VLC across 12 SSA countries for children and adolescents in 2020. However, results were variable for different countries reflecting the diversity among countries as most countries demonstrated an increase in VLC. Declines in VLC for countries with larger populations including South Africa, Kenya and Ethiopia were responsible for the overall VLC decrease among children. An additional two countries, Tanzania and Cote d’Ivoire also reported declines in VLC for adolescents. The differences between countries could possibly relate to variation in interruption of HIV services, the magnitude and impact of COVID-19 and interventions implemented. South Africa reported the highest number of COVID-19 cases and deaths in Africa [22]. Ethiopia reported an increase in deaths in 2020 and a significant decrease in the number of children with HIV newly started on ART [23]. Most countries saw an increase in VLC during December 2020 (Table 2). The moderate increase seen by October- December 2020 could potentially have been influenced by easing of restrictions, adaptations and broadening of differentiated community-based HIV services (including sample collection) implemented in the face of challenges related to COVID-19 [24,25]. These findings align with a stringency index from Oxford University which reported South Africa, Kenya, Ethiopia, Côte d’Ivoire and Lesotho reached a composite measure of at least 80 out of 100 [26]. Overall, there was a decline in the number of children eligible for VL testing while the number of eligible adolescents increased. This can be attributed to the disproportionate number of deaths in children compared to adolescents (27). In PEPFAR programs during fiscal year 2023, it is reported that the number of CLHIV starting and continuing ART is decreasing [27]. The fact that the prevention of mother to child HIV transmission (PMTCT) program is becoming increasingly effective leading to improvements in PMTCT coverage, and subsequently decrease in the number of children born with HIV may explain the observed decrease in the number of children currently on treatment [5].

Similar to findings from a recent study on effects of the pandemic in PEPFAR SSA countries, the number of patients with suppressed VL increased for the majority of countries for both children and adolescents improving upon pre-COVID-19 VLS gains [28]. Substantial progress has been achieved by country programs with support from PEPFAR and other stakeholders for scale up of HIV VL and progress toward epidemic control [29,30].

For the period covered by this analysis, the number of children with a documented VL increased for the majority of countries demonstrating resilience as countries managed the pandemic while continuing the HIV response. The decrease in total number of children with a documented VL occurred for South Africa, Kenya, Ethiopia, Cote d’Ivoire and Lesotho. Adolescents experienced a decrease in the same countries except for Lesotho which had a slight improvement. This decrease may be explained by COVID-19 related lockdowns impacting transportation, reduced number of patients’ clinic visits, and health care facility closures, especially at the beginning of the pandemic [2,12,15]. Equipment procured for HIV VL testing and personnel trained in molecular diagnostics were reallocated from HIV to SARS-CoV-2 testing [31]. Supply chain interruptions associated with border closures and flight restrictions decreased the availability of commodities including ART, medical, and laboratory supplies necessary for VL testing [15,32]. Reluctance of parents and caregivers to visit health facilities, being fearful of COVID-19 illness as reported in Ethiopia could also limit access to VL testing [23]. Although a direct cause and effect to VLC and suppression cannot be assessed for countries which experienced declines, the timing suggests an impact resulting from the pandemic, a hypothesis supported by multiple studies [15,33,34].

Adolescents may have traveled to VL testing facilities independently of adults, explaining the increase in the number of VL tests performed for adolescents compared to children [35]. Another factor could be that adolescents were less vulnerable than adults to critical illness, and may have had less fear to access medical care [36]. Some HIV programs embraced virtual platforms and use of electronic programs (e.g., WhatsApp) to increase ART adherence and monitoring [37]. Adolescents are likely to have better access to electronic devices compared to children. PEPFAR programs made adaptations to implement and to scale differentiated service delivery models that empower adolescents to take charge of their health, including remote counseling, ART optimization and adherence to clinic appointments [12,38,39].

Improvement in the number of children and adolescents with suppressed VL could in part be attributed to national policy guidance adaptations to HIV service delivery including multi-month dispensing, advocated in Côte d’Ivoire, DRC, Ethiopia, Kenya, Mozambique South Africa, Tanzania, Zambia and Zimbabwe [13]. Zambia reported home delivery for ART clients [40]. Multiple countries implemented community measures in response to facility-based closures to increase access to ART including community-based ART distribution, use of local pharmacies, and collection of samples for testing within the local community [12,37,41–43].

PEPFAR programs continued to implement ART optimization, specifically transitioning patients to DTG-based regimens while phasing out nevirapine and efavirenz to improve VL suppression outcomes for C/ALHIV [25,44,45]. Although roll out of DTG10 for younger children <20kg did not occur until 2021, roll out of DTG50 was underway in 2020 for children >20kg—<30kg. The increase in the number of children experiencing VLS (9 countries) and adolescents experiencing VLS (10 countries) suggests those who had access to ART were adherent as VLS is more likely among clients who are consistently adherent to ART [46]. C/ALHIV who were able to get a VL test, were possibly more adherent and/or had better access to health care services contributing to the observed increase in VLS. C/ALHIV who have not had a VL test may be among those least likely to be suppressed. HIV programs also collaborated with orphans and vulnerable children programs to support adherence and targeted interventions for the unsuppressed child/caregiver pair and adolescents [12]. Results by gender revealed minimal differences in the number of boys and girls eligible for a VL test, number with VL test results, number virally suppressed as well as VL coverage for both children and adolescents. However, adolescent boys and girls had a higher percentage change across all indicators compared to children. Countries such as Cameroon, DRC, Lesotho, Zambia and Zimbabwe are making progress on VLC and VLS among adolescents while there has been less progress for children between the two reporting periods. VLS increases were higher in 8 countries (Cameroon, Cote d’Ivoire, Kenya, Lesotho, Nigeria, Tanzania, Zambia and Zimbabwe) per Table 1. These findings highlight the existing gap for boys and girls lagging behind adolescents and adults. It is critical for programs to prioritize interventions for HIV diagnosis, ART and VL testing for children to document viral suppression [47]. Countries should consider implementing innovative strategies such as community-based monitoring, use of point-of-care technology and dried blood spots as an alternative to plasma to improve VLC for children [48].

There were some limitations to this analysis. Data used were collected quarterly thus month-to-month changes could not be assessed. Data on ART regimens for C/ALHIV were not available during the reporting periods and PEPFAR does not provide individual level data therefore, the impact of patients transition to DTG on VLS could not be determined. Another limitation is that PEPFAR DRC supports only three out of 25 total provinces. For the remaining 11 countries PEPFAR supports the majority of all C/ALHIV currently on ART [86%; range, 65% - 100%], supplemental Table 1 [6,14]. Due to country differences in HIV and the COVID-19 pandemic, countries implemented various COVID-19 mitigation measures, at different times and for different durations. Additionally, 2021 data was not evaluated, so longer term effects (direct /indirect) were not assessed. This analysis does not account for patient loss or aging in and out within pediatric and adolescent age-bands during the six months between reporting periods. As patients age in and out of these groups, the number of individuals currently on ART and those eligible for VL testing and coverage could change affecting the overall results.

## Conclusions

Achievement of the UNAIDS third 95 target in C/ALHIV during a dynamic and unpredictable pandemic, is an immense global health challenge, particularly in SSA where resources are limited [12]. Innovative research and approaches to combat dual epidemics in the future and patient-centered interventions to improve VLC and VLS among C/ALHIV are needed. Valuable lessons learned could be applied to future emerging infectious disease outbreaks. This study highlights the importance of collaborative efforts to improve health outcomes for C/ALHIV while addressing pandemic response.
